# Evaluation of the Swallow-Tail Sign and Correlations of Neuromelanin Signal with Susceptibility and Relaxations

**DOI:** 10.3390/tomography7020010

**Published:** 2021-03-27

**Authors:** Tzu-Wei Lee, Cheng-Yu Chen, Kuan Chen, Chao-Wei Tso, Hui-Hsien Lin, Ying-Liang Larry Lai, Fei-Ting Hsu, Hsiao-Wen Chung, Hua-Shan Liu

**Affiliations:** 1 School of Biomedical Engineering, College of Biomedical Engineering, Taipei Medical University, Taipei 110, Taiwan; superchrisxxx@gmail.com (T.-W.L.); jc985137@gmail.com (K.C.); dannytuzoo@gmail.com (C.-W.T.); 2 Department of Medical Imaging, Taipei Medical University Hospital, Taipei Medical University, Taipei 110, Taiwan; sandy0932@gmail.com; 3 Department of Radiology, School of Medicine, College of Medicine, Taipei Medical University, Taipei 110, Taiwan; 4 Translational Imaging Research Center, Taipei Medical University Hospital, Taipei 110, Taiwan; 5 CT/MR Division, Rotary Trading Co., Ltd., Taipei 115, Taiwan; twwarcgogo@gmail.com; 6 Ph.D. Program for Neural Regenerative Medicine, College of Medical Science and Technology, Taipei Medical University and National Health Research Institutes, Taipei 110, Taiwan; larrylai4800@gmail.com; 7 Department of Biological Science and Technology, China Medical University, Taichung 406, Taiwan; sakiro920@gmail.com; 8 Department of Electrical Engineering, National Taiwan University, Taipei 110, Taiwan; chunghw@ntu.edu.tw; 9 International Ph.D. Program in Biomedical Engineering, College of Biomedical Engineering, Taipei Medical University, Taipei 110, Taiwan

**Keywords:** susceptibility-weighted imaging, nigrosome-1, swallow-tail sign

## Abstract

The presence of a swallow-tail sign in the nigrosome-1 with hyperintense signal shown on the susceptibility-weighted imaging (SWI) has been shown to be sensitive in detecting the abnormal iron deposits in this area. A systematic evaluation in healthy subjects is required before this tool can be recommended in a widespread application. We evaluated a simple and practical SWI approach using a multiecho gradient-echo sequence with an improved contrast-to-noise ratio (CNR). We also evaluated the association of the neuromelanin imaging contrast behavior with the susceptibility and relaxation measurements. Twenty-five older and 23 young healthy adults were evaluated. The CNRs of the nigrosome-1 were compared along with method and group. Correlations of the nigrosome-1 neuromelanin signal in the neuromelanin-sensitive imaging with CNRs in the susceptibility, T1 and T2 mappings were examined. Two different coils were used to confirm the reproducibility. Compared with the single-echo, multiecho SWI can improve the CNR of the swallow-tail sign. We found significant correlations between neuromelanin signal and CNRs in the susceptibility and T2 mappings, and T1 value. The older subjects exhibited increased CNRs compared with the young adults. No significant difference was observed in the measurements between 20 and 64 channels. The multiecho technique allows the high-quality nigrosome-1 images in SWI and allows for a joint analysis of T2* and quantitative-susceptibility mapping at high spatial resolution. The correlations of neuromelanin-sensitive imaging with susceptibility and T2 imply that the iron content in the nigrosome-1 may have significant influences on the hyperintensity of neuromelanin in the magnetization transfer-related contrast.

## 1. Introduction

Nigrosome-1 has been identified as a swallow-tail sign with a small and hyperintense region in the substantia nigra (SN) on T2*-weighted or susceptibility-weighted imaging (SWI) sequences [1]. Nigrosome-1 abnormalities such as patients with Parkinson’s disease (PD) and Lewy body dementia exhibit an increased iron deposition in the pars compacta of the substantia nigra (SNpc) and the feature of swallow-tail sign is lost [2,3]. However, most reports have focused on the studies in patients with PD, few studies have evaluated the swallow-tail sign in healthy subjects. For swallow-tail sign imaging to become a useful clinical tool, a reliable technique which is reproducible and easy to use in the clinical environment is required to test the performance in healthy subjects before the tool can be recommended. This assessment can allow accurate delineation of the swallow-tail sign to improve the accuracy of tracking disease severity-related changes.

In addition to a single-echo sequence, SWI data can be acquired using a multiecho gradient-echo sequence, which can provide increased flexibility in selecting TEs, leading to an increase in the signal-to-noise ratio (SNR) and contrast-to-noise ratio (CNR) [4]. T2* and quantitative susceptibility mapping (QSM) have been reported to be sensitive in detecting the SN iron content when evaluating the pathophysiological processes such as PD progression [1,5]. A multiecho gradient-echo sequence which allows the diagnostic gain of T2* and QSM can thus be a potential prerequisite for clinical acceptance. However, most SWI examinations in clinical use are still confined to the conventional single-echo gradient-echo method.

T1-weighted neuromelanin-sensitive MRI (NM-MRI) can reflect the changes of neuromelanin and has shown the potential to detect alterations to SNpc morphology for PD [6]. The SN hyperintensity in the NM-MRI has been associated with the paramagnetic T1-shortening and magnetization transfer (MT) effects by the melanin-iron complex [7,8]. The presence of iron in this complex can also affect the transverse relaxation time [6]. However, limited data exist describing the relationship between hyperintensities of SNpc in the NM-MRI and its MR relaxation signals for healthy subjects.

In this study, we evaluated the multiecho SWI technique in the delineation of the swallow-tail sign through CNR measurements for healthy subjects. To investigate the possible association between the susceptibility of iron content and neuromelanin signal in the NM-MRI, we conducted exploratory analyses of correlations between MR signal in the neuromelanin with mappings of T1, T2 and quantitative susceptibility.

## 2. Materials and Methods

### 2.1. Participants

This study was approved by the local Institutional Review Board. Twenty-five healthy older (mean age 50.28 ± 12.15 years) and 23 young subjects (mean age 20.74 ± 1.21 years) without any history of neurological, cardiovascular, or other diseases were recruited (Table 1). One 73-year-old male patient who was diagnosed with PD since 2003 was also included for evaluation. Written informed consent was obtained from the participants. All the subjects were scanned using the 64-channel receiver array coil. To confirm the reproducibility of CNR measurements for the multiecho SWI_ave_ with a different coil, a total of 18 of the 23 young subjects were scanned for a second time by using the 20-channel coil, with the interval between scans ranging from 7 to 14 days.

### 2.2. Imaging Protocols

The data were acquired using a 3T (Magnetom Prisma, Siemens Healthcare, Erlangen, Germany) scanner. The SWI data were acquired using a three-dimensional (3D) multiecho gradient-echo sequence with the parameters: flip angle (FA) = 15°; TR = 51 ms; flow compensation of the first echo; imaging resolution = 0.58 × 0.58 × 1.5 mm^3^; and acceleration factor = 2. Eight echoes with TE ranging from 7.87 to 44.83 ms and an interval of 5.28 ms were acquired using unipolar readout gradients. For the comparison of the CNRs, SWI data acquired with a single-echo scan under the same spatial resolution (TR/TE = 35/25 ms, FA = 15°) were included.

For SWI, multichannel complex data were combined for optimal phase reconstruction and filtered using a high-pass homodyne filter to eliminate low-frequency phase variation [4,9]. A positive mask was generated using the filtered phase image. The generated mask was then multiplied with the corresponding magnitude image of the fifth power to obtain the final SWI for each echo. For multiecho SWI, based on the method proposed by Denk and Rauscher [4], the averaged susceptibility-weighted image (SWI_ave_) was generated by averaging the individual SWI from different echoes. The averaged magnitude (Mag_ave_) and unwrapped phase images (Pha_ave_) from different echoes in multiecho, and the magnitude (Mag) and unwrapped phase (Pha) images from the single-echo sequence were also included for the subsequent region-of-interest (ROI) analyses.

For QSM, phase data were unwrapped using a Laplacian-based approach, with the background field removed using the projection-onto-dipole-field method [10]. The susceptibility was obtained from the local tissue phase by using the morphology-enabled dipole inversion method [11]. T2* maps were computed from the eight magnitude images through nonlinear curve fitting of a monoexponential equation S(TE) = S_0_e^−TE/T2*^ by using the Levenberg–Marquardt least squares method. High-resolution T1-weighted anatomical images were collected by using a 3D MPRAGE sequence in the axial plane (voxel size of 0.9 × 0.9 × 0.9 mm^3^). The T1-weighted image was used as an anatomical reference that SWI acquisitions were oriented perpendicularly to the fourth ventricle floor [12].

To examine how NM-MRI signal related to susceptibility and relaxation times in the nigrosome-1, we acquired data of NM-MRI, T1 and T2 mappings for young subjects using 64-channel array coil. T1-weighted fast spin-echo sequence was used to acquire NM-MRI: TR/TE = 787/13 ms; echo train length = 3; section thickness = 2.5 mm with no gap; 13 slices (voxel size of 0.54 × 0.54 × 2.5 mm^3^); NEX = 5. T1 mapping was performed using a multiple flip-angle gradient-echo sequence with TR/TE = 20/5.3 ms, α = 5°, 10°, 15°, 20°, 25°, 30°, resolution = 0.6 × 0.6 × 1.5 mm^3^. T1 maps were calculated by fitting the Ernst equation with B1 correction [13]. T2 mapping was performed using a multiple-echo TSE acquisition with TR = 4530 ms, TE ranging from 13.8 to 124.2 ms with an interval of 13.8 ms and α = 90° (voxel size of 0.6 × 0.6 × 1.5 mm^3^). T2 maps were calculated using the scanner manufacturer-supplied software.

### 2.3. Imaging Data Analyses

**Figure 1:**
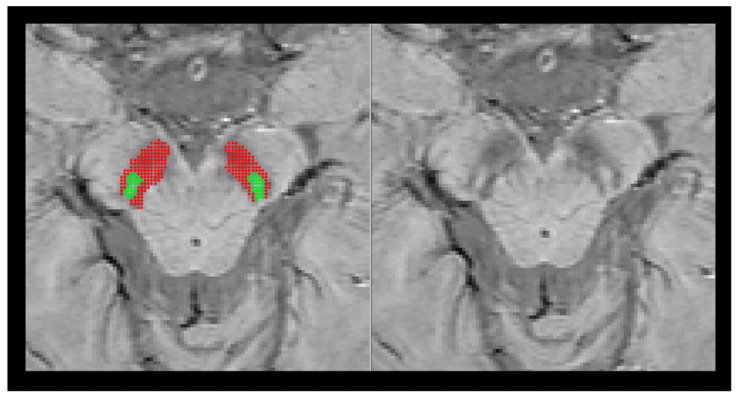
Left: ROI placement in the SWI map. A ROI was first manually selected on the substantial nigra according to SWI_ave_. The fuzzy c-means clustering (FCM) algorithm was then applied on the selected ROI to identify the first cluster class with hyperintense signals representative of the nigrosome1 structure (green dots) and the second cluster class with hypointense signals representing the substantia nigra structures adjacent to the nigrosome1 structure (red dots). Right: The original SWI map without ROI overlap.

Coregistration between the SWIs, NM-MRI, T2 and T1 mappings was performed using software (Statistical Parametric Mapping, version SPM8). Because there are few slices for neuromelanin images, reorienting the NM images to match with the SWI as close as possible can provide better coregistration. First, all of the images were reoriented by using the interpeduncular fossa (supplementary Figure S1) as a landmark for the origin of the coordinate system so those images can be properly aligned with each other. Next, neuromelanin images along with other images were coregistered with multi-echo SWI_ave_ using Statistical Parametric Mapping 12 software. Finally, an image overlay of the segmented SN ROIs was produced from the SWI_ave_ and other imaging data to check the quality of registration (supplementary Figure S2). The quality of registration was checked by visual inspection. Manual adjustment was performed using the interactive tool of manual registration in ITK-SNAP [14] if there is a presence of coregistration error. A ROI was first manually selected on the SN in each slice containing the swallow-tail sign according to the multiecho SWI_ave_ because the best imaging quality was determined from SWI_ave_. ROI analyses of the images were performed by two experienced researchers (H-S L and T-W L), who were blinded to subject information, under close supervision of a senior author (C- Y C, board-certified neuroradiologist with more than 20 years of experience in neuroimaging). The interobserver reliability was assessed by calculating intraclass correlation coefficients with a *p* value < 0.005. The two reviewers defined the margins of the SN on each axial plane containing the swallow-tail sign through consensus. The authors manually outlined the margins of SN on the SWI_ave_, in which the SN area shows a good definition of the hypointense boundaries. An automatic segmentation method based on a two-class fuzzy c-means clustering (FCM) algorithm was then applied to remove the possible intraobserver variation [15]. The first cluster class with hyperintense signal was identified and representative of the nigrosome-1 structure and the second cluster class with hypointense signal representing the substantia nigra structures adjacent to the nigrosome-1 structure (Figure 1). Manual adjustment of image segmentation was performed if necessary. These ROIs were then applied bilaterally in the SWI_ave_, Mag_ave_, Pha_ave_, QSM, T2*, T2, T1, SWI, Mag, and Pha of every subject. Image processing was conducted through an in-house MATLAB (MathWorks, Natick, MA) pipeline.

**Figure 2:**
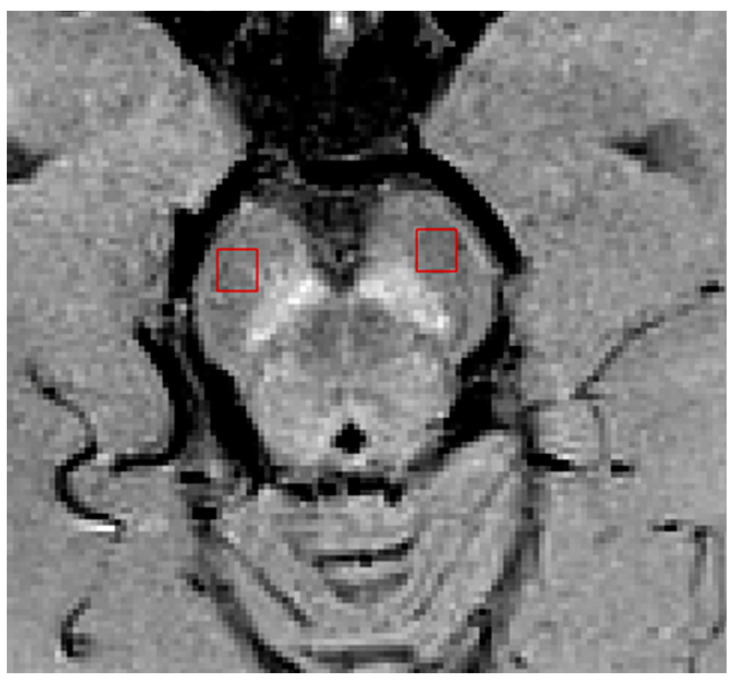
Definition of regions of interest (ROIs) for cerebral crus (red).

The CNR was calculated using the formula: CNR = (S_N1_ − S_0_)/σ_0_, where S_N1_ and S_0_ refer to the mean signals within the nigrosome-1 and the neighboring substantia nigra ROIs, respectively, and σ_0_ refers to the standard deviation of the neighboring substantia nigra ROI. The neuromelanin signal of the nigrosome-1 in NM-MRI from the above-mentioned ROI of nigrosome-1 was normalized to the reference value of the mean signals within the background ROI defined in the cerebral crus (Figure 2) [16]. Because we did not observe any significant difference between the left and right sides for all the measurements (*p* > 0.05), the mean values of the bilateral measurements were used for the subsequent statistical analyses.

### 2.4. Statistical Analysis

The age effect was evaluated using the generalized linear model with group (older vs. young groups), sex, and group × sex as the independent variables. The Chi-squared test or Fisher’s exact test was used for comparing categorical variables. Linear mixed-effect (LME) regression modeling was used to compare the CNRs obtained for the two groups from different MR methods. In the LME procedure, the CNR of the swallow-tail sign was the dependent variable and the various MR methods were the independent variables. The MR method was the within-subjects factor, and the study group was the between-subject variable. Group differences between the older and young subjects in the quantitative measurements of QSM, T2* and phase in the nigrosome-1 and neighboring SN area were assessed with Wilcoxon signed-rank tests. For comparing different receiver coils, the Wilcoxon signed-rank tests were used to evaluate the difference between the 20- and 64-channels in the measurement of single- and multiecho SWIs for the young subjects. Spearman correlations were used to examine whether nigrosome-1 neuromelanin signal in NM-MRI was associated with CNRs and quantitative values of QSM, T2 and T1 mappings. Because we found the significant correlations between NM-MRI signal and CNRs of QSM and T2 mapping in the nigrosome-1, an exploratory Spearman correlations was further conducted to exam the associations between quantitative measurements of QSM and T2 in the nigrosome-1. A *p*-value of 0.05 was considered significant. The SPSS software version 20.0 (IBM, Armonk, NY, USA) was used to conduct the analyses.

## 3. Results

**Figure 3:**
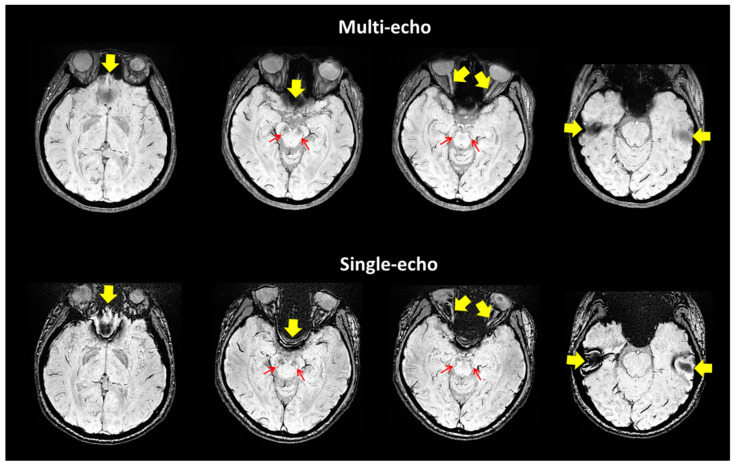
Representative images of multiecho (upper row) and single-echo SWIs (lower row) from a 38-year-old female healthy subject with four slices. Multiecho SWI exhibits improved image quality with increased SNR and CNR in the nigrosome-1 (arrows) and reduced susceptibility artifacts in observing the optic nerves and frontal and temporal lobes (arrow heads).

**Figure 4:**
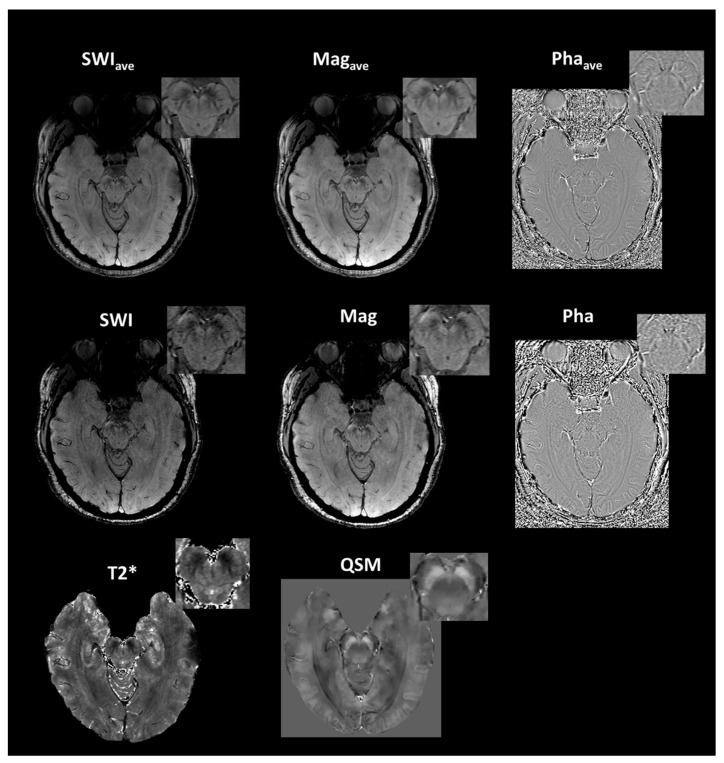
Representative images of susceptibility-weighted (SWI_ave_), magnitude (Mag_ave_), and phase (Pha_ave_) imagings acquired using the multiecho method (first row) and single-echo method (second row, from left to right, SWI, magnitude and phase imagings) as well as the T2* and quantitative-susceptibility mappings (QSM, third row) for a 20-year-old female healthy subject.

**Figure 5:**
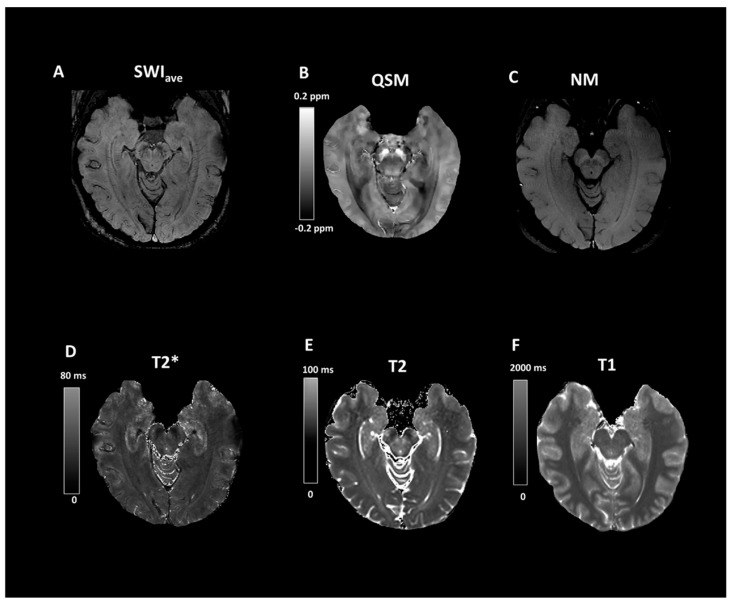
Representative images of (**A**) multi-echo susceptibility-weighted image (SWI_ave_), (**B**) quantitative-susceptibility mappings (QSM), (**C**) neuromelanin-sensitive MRI (NM-MRI), (**D**) T2* map, (**E**) T2 map and (**F**) T1 map for a 20-year-old female healthy subject.

**Figure 6:**
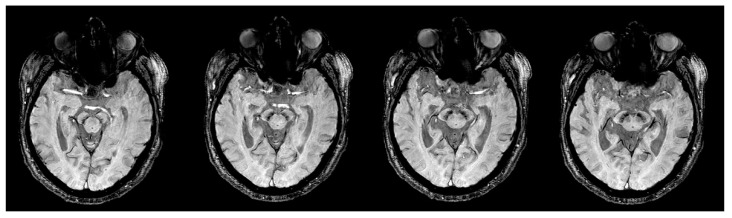
Four consecutive slices of the multi-echo susceptibility-weighted imaging from a 73-year-old male patient with Parkinson’s disease. No swallow-tail sign is observed in the substantia nigra areas.

Table 1 summarizes the demographic information of the study cohorts. There is a significant age difference between two groups (F = 129.164, *p* < 0.0001). No other demographic differences were observed. Figure 3 depicts the representative SWI comparison between single-echo and multiecho SWI techniques. The phase variation artifacts in the frontal and temporal lobe areas were successfully improved using the multiecho SWI_ave_ technique. The SWI_ave_ technique further exhibited a superior imaging quality in visualizing the optic nerve. Figure 4 illustrates a representative slice from a single subject in the native space of the QSM, T2*, SWI_ave_, and SWI images with their corresponding phase and magnitude images acquired from multiecho and single-echo sequences, respectively. Table 2 lists the results of the CNRs obtained using the different imaging methods. The corresponding quantitative measurements of the phase, T2*, and QSM in nigrosome-1 and neighboring SN area for each subgroup are presented in Table 3. Figure 5 shows a representative slice from a subject in the native space of the NM-MRI, QSM, T1 and T2 mappings. The mean relaxation times were T1 = 1226.99 ± 221.17 ms and T2 = 81.18 ± 5.63 ms for the nigrosome-1; T1 = 1230.81 ± 238.56 ms and T2 = 75.79 ± 7.43 ms for the neighboring SN area, respectively. We further attached the imaging of multiecho SWI_ave_ for a patient with PD for comparison (Figure 6). It is obvious that the swallow tail sign is absent for this PD patient as expected.

**Figure 7:**
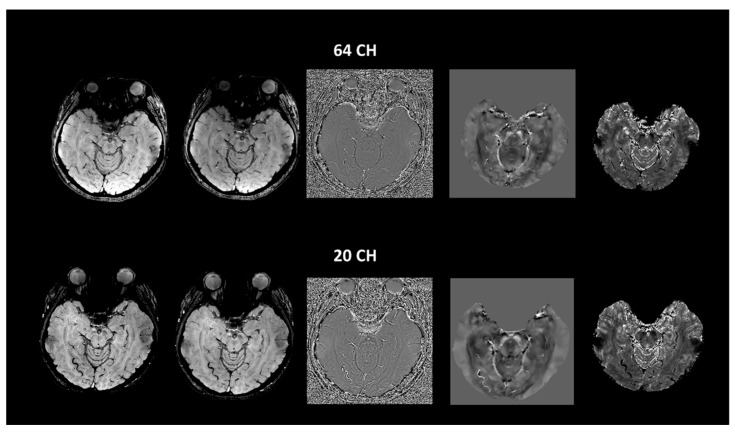
Comparison of the images acquired using the multiecho gradient method with 64- (upper row) and 20-channel (bottom row) receiver coils in SWI, magnitude imaging, phase imaging, QSM, and T2* imaging (from left to right) for a 20-year-old female.

Linear regression analyses comparing the CNR values obtained between the two groups using different methods indicated the considerable influence of the selected method (F = 902.896, *p* < 0.0001) and the group (F = 30.076, *p* < 0.0001). Post-hoc pairwise comparisons indicated that the older group exhibited a considerably higher swallow-tail CNR than the young group when using the SWI_ave_ (*p* = 0.003), Mag_ave_ (*p* = 0.007), SWI (*p* = 0.004), Mag (*p* = 0.005), and T2*(*p* = 0.008) methods. SWI_ave_ demonstrated the most significant group difference as compared with other methods. For both the older and young groups, the SWI_ave_ method generated the highest CNR. On average, the SWI method exhibited a higher CNR of the swallow-tail sign compared with the corresponding magnitude, phase, T2*, and QSM methods (Table 2). Moreover, we found that compared with the young subjects, the older subjects exhibited considerably higher mean values of the quantitative measurements of QSM in the nigrosome-1 (*p* < 0.001) and neighboring substantia nigra (*p* < 0.001) (Table 3). For the comparison of different coils, we did not find any significant difference in all of the CNR measurements between the 20- and 64-channel array coils (Figure 7, *p* > 0.05).

**Figure 8:**
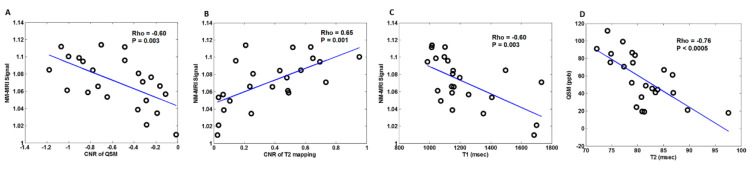
Scatter plots show significant correlations of neuromelanin signals in NM-MRI with (**A**) CNRs in QSM, (**B**) CNRs in T2 mapping and (**C**) T1 values in the nigrosome-1. (**D**) Significant correlation of quantitative measurements between QSM and T2 values in the nigrosome-1.

There were no correlations of NM-MRI signal with quantitative values of susceptibility and T2 in the nigrosome-1(*p* > 0.05). However, significant correlations between neuromelanin signal in NM-MRI (normalized to cerebral crus) and CNRs in QSM (Rho = −0.60, *p* = 0.003) and T2 mapping (Rho = 0.65, *p* = 0.001) of the nigrosome-1 were identified. We also found a significant correlation between neuromelanin signal and T1 values in the nigrosome-1 (Rho = −0.60, *p* = 0.003) (Figure 8). An exploratory analysis further showed a significant correlation between QSM and T2 values in the nigrosome-1(Rho = −0.76, *p* < 0.0005) (Figure 8).

## 4. Discussion

This study was conducted to assess the potential of using a simple and practical method of multiecho SWI_ave_ in the delineation of the swallow-tail sign of nigrosome-1 and compare with the single-echo SWI which is the most commonly used in routine clinical practice [2,17]. The major findings of this study are: first, compared with the conventional single-echo SWI, the simple method using multiecho SWI_ave_ can improve the CNR of the swallow-tail sign; second, significant correlations between neuromelanin signal and CNRs in QSM and T2 mapping, and T1 value of the nigrosome-1 were identified; third, the older subjects exhibited increased CNR values compared with the young adults.

Seeking a novel approach through a multiecho gradient-echo sequence to map for the evaluation of nigrosome-1 has been carried out in various studies [12,18,19]. MR transverse relaxation and QSM have also been employed to characterize iron deposition within the SN in the diagnosis of PD [20]. QSM is theoretically more accurate in iron quantification and has attracted considerable interest as a potential means of quantifying neuronal loss and iron accumulation in the SNpc for patients with PD [21,22]. However, our results demonstrated that SWIs yielded a superior and distinct CNR in visualizing the anatomical details of the swallow-tail sign in the diagnosis of the nigrosome-1 compared with QSM and T2*. QSM is more computationally expensive since it requires several processing steps, including phase unwrapping, brain extraction, background phase removal and overcoming the ill-posed problem [10]. An accurate estimation of the QSM is thus essential for approaches using QSM [21] or QSM-generated mask [18,19] in detecting the swallow-tail sign. This may need computational expertise, especially given that QSM has not been widely used for clinical routine as compared with SWI so far. Nigrosome-1 is hyperintense in SWI because of its low iron content than the surrounding tissues [23]. The multiecho SWI_ave_ method in the present study is relatively simple and practical for clinical use with less computationally demanding and can provide much higher CNR than QSM (more than three times) and conventional single-echo SWI in detecting the swallow-tail sign. In a multiecho gradient-echo sequence, because several echoes can be collected within one TR, the acquisition time can be potentially not prolonged, while the SNR can increase [4]. The considerably improved CNR with the SWI_ave_ technique shown in the present study could be attributed to the increased SNR.

It is worth noting that both QSM and phase images displayed negative values of the CNR in nigrosome-1 compared with other imaging methods. These results provide evidence that nigrosome-1 in the SNpc is less paramagnetic than the surrounding tissues, such as substantia nigra pars reticulate (SNpr) in which the brain region appeared to have a high iron content. The less paramagnetic susceptibility of the nigrosome-1 than the surrounding tissues could be resulted from its known low iron content than the surrounding tissues as mentioned above [23]. This is further supported by the observation that the neighboring SN area exhibited lower T2* values than the nigrosome-1 in the quantitative T2* data analysis in our study (Table 3).

The source of NM-sensitive contrast has been suggested as a combination of MT and T1-shortening effects, in which the latter is associated with the paramagnetic nature of melanin-iron complex [24]. The neuromelanin pigment has been reported to act as a T1-shortening agent when bound with iron molecules [7,25]. Our observation of negative correlation between the NM-MRI signal and T1 values added support to this speculation (Figure 8C). We found a significant correlation between NM-MRI signal and CNR of T2 mapping in the nigrosome-1 (Figure 8B). It has been reported that T2 values tend to increase as the size of paramagnetic perturbers increases and melanin-iron complex is known to have a significantly larger effective size as compared with the ferric iron [26]. The observed positive correlation between these two measures may be associated with the quantity of the melanin-iron complex-containing neurons in the nigrosome-1, in which the increased signal intensity of the NM-MRI and CNR in the T2 mapping could be attributed to the increased melanin-iron content in this brain region. This finding also coincides with the previous report that the presence of iron in the melanin-iron complex can affect the proton transverse relaxation time [6], which is also supported by the result of significant correlation between susceptibility measurements (i.e., QSM) and T2 values of the melanin-iron complex in the nigrosome1 (Figure 8D). Due to the problem of magic angle in QSM, the calculated susceptibility values are therefore relative rather than absolute quantification [27]. We speculate that this quantification error along with the one in calculation of the absolute T2 values may lead to the limited sensitivity in correlating with the neuromelanin signal. Our finding may imply that CNR preserves the information of the relationship between neuromelanin signal, the content of paramagnetic susceptibility and T2 relaxation for the melanin-iron complex-containing neurons in the nigrosome-1. We also observed a significant correlation between NM-MRI signal and CNR of QSM in the nigrosome-1 (Figure 8A). The correlation between these two measures implies that the content of paramagnetic susceptibility in the nigrosome-1 have significant influences on the hyperintensity of SNpc in the T1 and MT-related contrast of NM-MRI.

We demonstrated the increased CNR of the swallow-tail sign in older subjects. This result is concordant with the age-related iron deposits for older subjects [28,29], especially in the SNpr, which is one of the brain areas that have high iron concentration. Exploratory measurements of QSM and T2* provide further evidence showing significantly higher paramagnetic susceptibility in the nigrosome-1 and the surrounding area in older subjects. The observed increased paramagnetic susceptibility of the nigrosome-1 in the older group was also corroborated by the known age effect of the gradual increase of NM [26], which is known with the paramagnetic nature of melanin-iron complex.

We ran the scans of CNR measurements in the swallow-tail sign using a 20-channel coil that had similar results to 64-channels. Although a comparison of 96- and 32-channel arrays has been reported in a previous study [30], no studies have compared the difference of swallow-tail sign between 64- and 20-channels in clinical practice. The more complementary information provided by the collection and analysis of different phased-array MR imaging data can enable more complete exploitation of current technologies using phased-array coils in clinical systems, especially for some clinical institutes using the 20-channel coil instead of 64-channel one as a routine practice. The comparison between the 64- and 20-channel coils revealed negligible differences of the CNR in the delineation of SNpc. It has been reported that the central SNR is nearly identical and the peripheral SNR is higher in the coil with a higher channel count compared with a lower channel one [30]. The sensitivity in the center for accelerated acquisitions can be affected only for more highly accelerated imaging [31,32]. We attributed our results to the similar SNR measurements in the central brain regions between different array coils because the acceleration (factor = 2) we used in the present study was not high.

Our study had limitations. First, even though the quality of registration was carefully checked by visual inspection with particular care to manually adjust the image registration if there is a presence of coregistration error, we cannot exclude possible confounds caused by the misregistration of the small size for nigrosome-1. Second, a 3D acquisition of several echoes with a relatively long scan time may still lead to practical limitations for subjects who may have difficulty maintaining a stable head position. Third, this study was conducted mainly in healthy subjects. Future studies of a prospective evaluation showing a comparison between subjects with PD and without PD are needed to investigate in detail and support the advantage of this technique.

In conclusion, we used a multiecho SWI_ave_ technique at 3T to demonstrate its advantages in obtaining the CNR and other quantitative information for delineating the nigrosome-1 of the swallow-tail sign. A multiecho gradient-echo sequence can also benefit from quantitative QSM and T2* measurements for assessing the brain iron content with the capabilities of SWI_ave_. Our results of correlations between CNRs in QSM, T2 mapping and NM-MRI provide a new understanding of the contrast behavior of the nigrosome-1, indicating that using these techniques may provide more comprehensive information of the melanin-iron complex in nigrosome-1.

## Figures and Tables

**Table 1:** Subject age and sex by groups.

Group (*n*)	Sex (*n*, %)	Age, Mean (SD)
Older adults (25)	Male (10, 40.0%)	50.28 (12.15) years
	Female (15, 60.0%)	
Young adults (23)	Male (11, 47.8%)	20.74 (1.21) years
	Female (12, 52.2%)	

**Table 2:** Mean and standard deviation (std) of the contrast-to-noise ratios in the nigrosome-1 of the swallow tail sign for different groups and methods.

			SWI_ave_	Mag_ave_	Pha_ave_	SWI	Mag	Pha	QSM	T2*
64 CH	Older	Mean (std)	2.75 (0.22)	2.03 (0.23)	−0.55 (0.23)	1.99 (0.32)	1.67 (0.29)	−0.44 (0.18)	−0.80 (0.39)	1.80 (0.44)
	Young	Mean (std)	2.52 (0.28)	1.77 (0.37)	−0.62 (0.35)	1.67 (0.36)	1.39 (0.34)	−0.46 (0.25)	−0.57 (0.34)	1.47 (0.49)
20CH	Young	Mean (std)	2.58 (0.30)	1.82 (0.30)	−0.60 (0.24)	1.70 (0.29)	1.41 (0.29)	−0.47 (0.20)	−0.61 (0.32)	1.49 (0.36)

64CH: 64-channel phased-array coil, 20CH: 20-channel phased-array coil, Older: older subject group, Young: young subject group.

**Table 3:** Quantitative susceptibility mapping (QSM, ppb), T2* (msec) and phase (radian) measurements in the nigrosome-1 (S_N1_) and neighboring substantia nigra area (S_0_).

			QSM		T2*		Pha_ave_	
			S_N1_	S_0_	S_N1_	S_0_	S_N1_	S_0_
64CH	Older	Mean (std)	90.20 (33.01)	136.99 (43.38)	36.02 (5.86)	25.26 (5.04)	−0.02 (0.01)	0.02 (0.02)
	Young	Mean (std)	53.16 (29.19)	85.52 (37.27)	38.86 (4.49)	28.94 (4.85)	−0.02 (0.01)	0.03 (0.02)
		*p*-value ^a^	<0.001 *	<0.001 *	0.55	0.13	0.29	0.57

64CH: 64-channel phased-array coil, 20CH: 20-channel phased-array coil, Older: older subject group, Young: young subject group, ppb: parts per billion. ^a^: Wilcoxon signed-rank tests of group comparison between older and young subjects using 64-channel phased-array coil; * *p* < 0.001, statistically significant.

## Supplementary Materials

The following are available online at https://www.mdpi.com/article/10.3390/tomography7020010/s1, Figure S1: The interpeduncular fossa (arrows) was used as a landmark for the origin of the coordinate system in the process of image coregistration so all of the images can be reoriented to be properly aligned with each other. The underlay images shown in the figure are the neuromelanin images; Figure S2. An image overlay of the segmented substantia nigra regions (the pars compacta and the neighboring area of the substantia nigra regions of interest were indicated by yellow and red colors, respectively) was produced from the SWIave and other imaging data to check the quality of final image registration. NM: neuromelanin image.
