# Supplementary material for: Evaluation of the Swallow-Tail Sign and Correlations of Neuromelanin Signal with Susceptibility and Relaxations
Source: Tomography. 2021 Mar 27;7(2):107–19. doi: 10.3390/tomography7020010 (PMC8103261; doi:10.3390/tomography7020010)
Supplement: Supplementary file 1 [file tomography-07-00010-s001.pdf]

Supplementary Figures

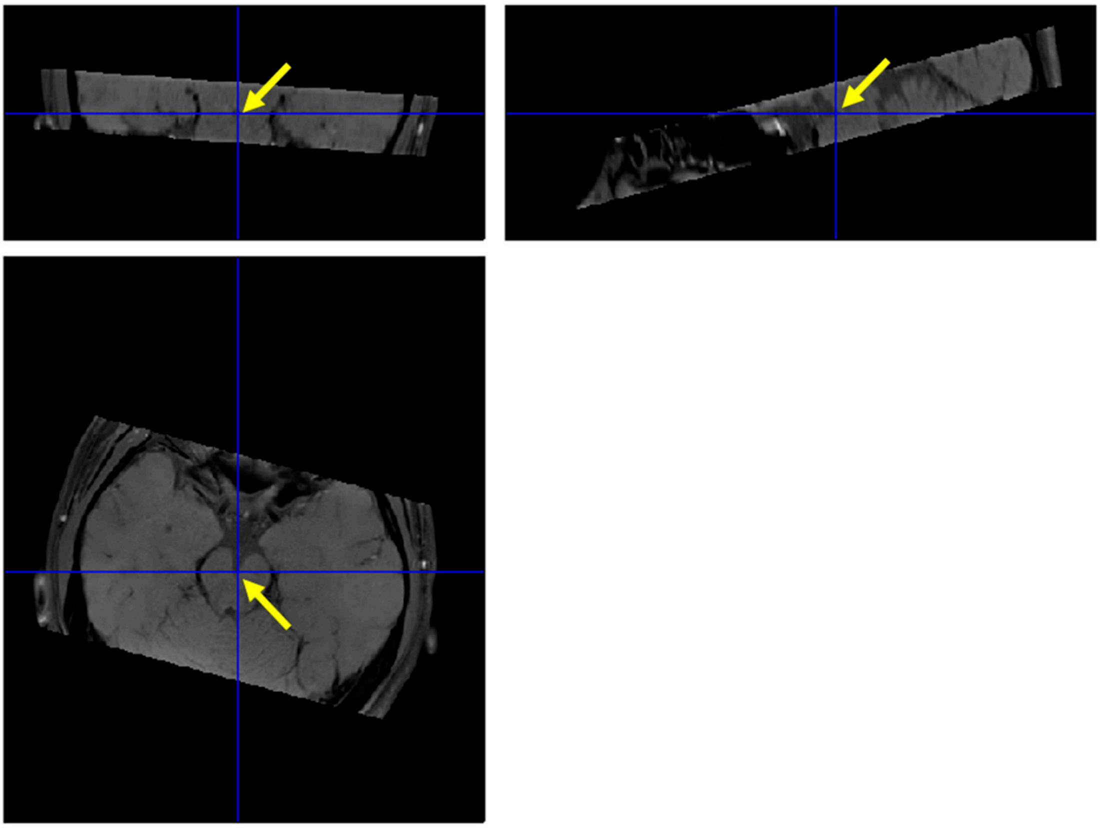

Figure S1. The interpeduncular fossa (arrows) was used as a landmark for the origin of the coordinate system in the process of image coregistration so all of the images can be reoriented to be properly aligned with each other. The underlay images shown in the figure are the neuromelanin images.

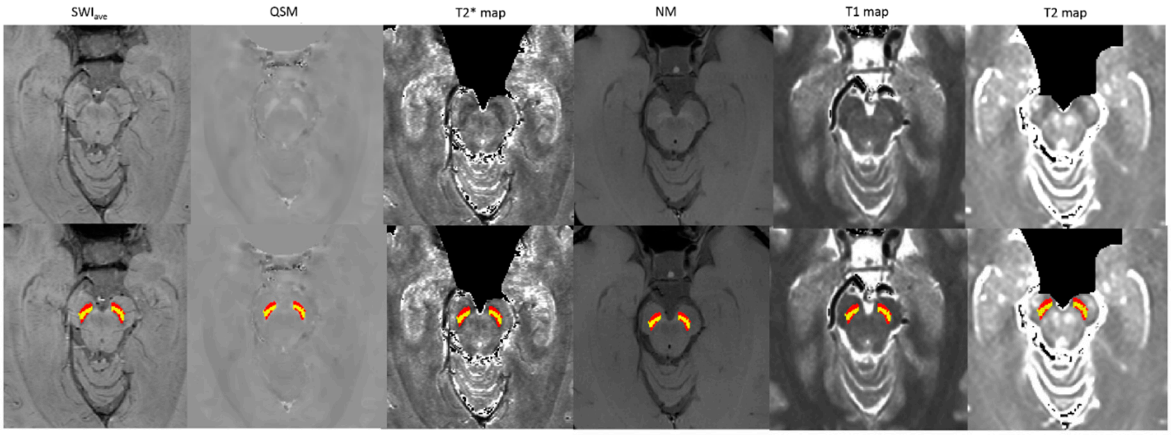

Figure S2. An image overlay of the segmented substantia nigra regions (the pars compacta and the neighboring area of the substantia nigra regions of interest were indicated by yellow and red colors, respectively) was produced from the SWI$_{ave}$ and other imaging data to check the quality of final image registration. NM: neuromelanin image.
